# How Does Blood-Retinal Barrier Breakdown Relate to Death and Disability in Pediatric Cerebral Malaria?

**DOI:** 10.1093/infdis/jiaa541

**Published:** 2020-08-26

**Authors:** Ian J C MacCormick, Valentina Barrera, Nicholas A V Beare, Gabriela Czanner, Michael Potchen, Samuel Kampondeni, Robert S Heyderman, Alister G Craig, Malcolm E Molyneux, Macpherson Mallewa, Valerie A White, Dan Milner, Paul Hiscott, Terrie E Taylor, Karl B Seydel, Simon P Harding

**Affiliations:** 1 Department of Eye and Vision Science, Institute of Life Course and Medical Sciences, University of Liverpool, a Member of Liverpool Health Partners, Liverpool, United Kingdom; 2 Malawi-Liverpool-Wellcome Trust Clinical Research Programme, Queen Elizabeth Central Hospital, Blantyre, Malawi; 3 Centre for Inflammation Research, University of Edinburgh, Edinburgh, United Kingdom; 4 NHS Blood and Transplant, Tissue and Eye Services R&D, Liverpool, United Kingdom; 5 St. Paul’s Eye Unit, Liverpool University Hospitals Foundation Trust, Liverpool, United Kingdom; 6 Department of Applied Mathematics, Liverpool John Moores University, Liverpool, United Kingdom; 7 Department of Imaging Sciences, University of Rochester Medical Center, Rochester, New York, USA; 8 Blantyre Malaria Project, University of Malawi College of Medicine, Blantyre, Malawi; 9 Division of Infection and Immunity, University College London, London, United Kingdom; 10 Liverpool School of Tropical Medicine, Pembroke Place, Liverpool, United Kingdom; 11 Department of Paediatrics and Child Health, Queen Elizabeth Central Hospital, Blantyre, Malawi; 12 Department of Pathology and Laboratory Medicine, University of British Columbia, Vancouver, British Columbia, Canada; 13 American Society for Clinical Pathology, Chicago, Illinois, USA; 14 Department of Osteopathic Medical Specialties, College of Osteopathic Medicine, Michigan State University, East Lansing, Michigan, USA

**Keywords:** blood-brain barrier, malarial retinopathy, brain swelling, cerebral malaria, fluorescein angiography

## Abstract

**Background:**

In cerebral malaria, the retina can be used to understand disease pathogenesis. The mechanisms linking sequestration, brain swelling, and death remain poorly understood. We hypothesized that retinal vascular leakage would be associated with brain swelling.

**Methods:**

We used retinal angiography to study blood-retinal barrier integrity. We analyzed retinal leakage, histopathology, brain magnatic resonance imaging (MRI), and associations with death and neurological disability in prospective cohorts of Malawian children with cerebral malaria.

**Results:**

Three types of retinal leakage were seen: large focal leak (LFL), punctate leak (PL), and vessel leak. The LFL and PL were associated with death (odds ratio [OR] = 13.20, 95% confidence interval [CI] = 5.21–33.78 and OR = 8.58, 95% CI = 2.56–29.08, respectively) and brain swelling (*P* < .05). Vessel leak and macular nonperfusion were associated with neurological disability (OR = 3.71, 95% CI = 1.26–11.02 and OR = 9.06, 95% CI = 1.79–45.90). Large focal leak was observed as an evolving retinal hemorrhage. A core of fibrinogen and monocytes was found in 39 (93%) white-centered hemorrhages.

**Conclusions:**

Blood-retina barrier breakdown occurs in 3 patterns in cerebral malaria. Associations between LFL, brain swelling, and death suggest that the rapid accumulation of cerebral hemorrhages, with accompanying fluid egress, may cause fatal brain swelling. Vessel leak, from barrier dysfunction, and nonperfusion were not associated with severe brain swelling but with neurological deficits, suggesting hypoxic injury in survivors.

## Teaser

Fluorescein angiography provides evidence that in cerebral malaria severe brain swelling and death are due to fluid egress from multiple small cerebral hemorrhages. In contrast, neurological deficits in survivors are associated with vessel leak and capillary nonperfusion.

After a period of global decline in malaria, progress has stalled with approximately 230 million cases and 405 000 deaths in 2018. Ninety percent are in sub-Saharan Africa, mainly children under 5 years [1]. *Plasmodium falciparum* causes several interrelated life-threatening syndromes in children: severe malarial anemia, metabolic acidosis, and cerebral malaria (CM). Cerebral malaria has a stubbornly high mortality rate of approximately 15% despite treatment in a specialist unit [2], but this can be higher in less well resourced settings [3]. As well as neurological sequelae at discharge, survivors can develop neurocognitive delay, epilepsy, and behavioral changes [4–6].

The development of new treatments requires a better understanding of pathogenesis for insights into improved supportive care [7]. This is hampered by the difficulty of studying the brain in vivo, and in malaria-endemic regions the challenge is magnified by lack of infrastructure and imaging.

Nevertheless, an important characteristic of pediatric CM is clear: interactions between parasitized red blood cells (pRBCs) and the microvascular endothelium (sequestration) evolve into severe pathology. This includes congestion and occlusion of capillaries and venules, inflammation, and dysregulation of local coagulation [8, 9]. Binding of pRBCs to endothelial protein C receptor (EPCR) has been associated with severe malarial disease, brain swelling, and blood-brain barrier (BBB) breakdown [10, 11]. Magnetic resonance imaging (MRI) studies have found that severe brain swelling is strongly associated with death [2]. In some patients, appearances are similar to posterior reversible encephalopathy syndrome, suggesting that BBB failure, combined with venous congestion, contributes to brain swelling [12]. Findings on susceptibility-weighted imaging in children with CM are consistent with venous congestion from sequestration, inflammation, and autoregulatory dysfunction [13].

The resolution of MRI is poor compared with retinal biomicroscopy. The effects of sequestration on capillaries in the retina can be visualized and are specific. The presence of malarial retinopathy on funduscopy in a comatose child with *P falciparum* improves the specificity of diagnosis [14–19]. Similarities between retina and brain suggest that retinal observations could provide insight into dynamic microvascular processes occurring throughout the central nervous system (CNS) and their relationship to severe brain swelling and death [17]. The densities of pRBC sequestration in retina and brain are correlated, and more severe retinopathy cases have more cerebral vascular congestion, mature parasites, and extraerythrocytic hemozoin [14]. Blood-retina barrier (BRB) function is dependent on endothelial cells and pericytes that are severely disrupted or lost in association with sequestration. Retinal fluorescein angiography (FA) utilizes intravenous fluorescein, a small, largely unbound molecule, to demonstrate retinal perfusion and also detect any BRB dysfunction. Fluorescein is an exquisitely sensitive marker of BRB breakdown through leakage.

Fluorescein angiography has revealed that funduscopic retinal vessel changes are due to intravascular filling defects and occlusion, demonstrated histologically to be due to sequestered pRBCs [15]. A preliminary study also showed several patterns of BRB breakdown, or leakage, in pediatric CM [20], but associations between retinal leakage, brain swelling, and death or disability are unknown. We investigated the contribution of BBB breakdown to severe brain swelling, death, and neurodisability in CM by examining the BRB using FA. Given the extensive similarities between retina and brain in pediatric CM, we hypothesized that leakage occurs proportionately in both retina and brain, and the effect of this leakage on mortality is mediated by severe brain swelling. We did not investigate FA as a prognostic predictor of outcome in CM.

## METHODS

### Study Design and Participants

We performed a prospective cohort study of FA features and clinical outcomes in children with retinopathy-positive CM. The participants were part of a research program in Queen Elizabeth Central Hospital, Blantyre, Malawi, in which a subset had admission brain MRI. Ocular tissue from children who had undergone autopsy was also available.

We defined pediatric CM according to World Health Organization criteria: *P falciparum* parasitemia, Blantyre Coma Score <3, and no other evident cause of coma [8]. This definition is broad and inevitably includes other conditions causing coma in endemic areas where asymptomatic parasitemia is common. We therefore limited our study to participants with malarial retinopathy [8, 18]. An ophthalmologist performed dilated indirect ophthalmoscopy and standardized grading [21]. Retinopathy was present if 1 or more of these signs were seen: retinal hemorrhage, retinal whitening, orange or white vessel discoloration [22].

Clinical outcome was determined at discharge as full recovery, recovery with neurological sequelae, or death. Neurological sequelae included any new neurological deficit evident on clinical examination [6].

Parents or guardians gave written informed consent. We adhered to the Declaration of Helsinki, and the ethics committees of University of Malawi College of Medicine and Michigan State University approved the study.

### Prospective Retinal and Brain Imaging Study

We recruited patients during the malaria seasons of years 2006 to 2014, excluding 2011 when FA was not performed. After clinical stabilization and dilated indirect ophthalmoscopy, patients underwent FA and brain MRI on the day of, or day after admission. Brain MRI was introduced in 2009. Patients were excluded if they did not have FA within this timeframe. Fluorescein angiography and MRIs were not performed if the child was clinically unstable or had rapidly resolving coma.

#### Retinal Imaging and Grading

An ophthalmologist took 50° color and FA images using a table-mounted camera (Nikon D1-H; Topcon TRC-50EX; Imagenet 2000; Topcon, Tokyo, Japan). Sodium fluorescein 20% was injected intravenously, dosed by weight (5–10 kg 2 mL; 11–20 kg 3 mL; 21–30 kg 4 mL; >30 kg 5 mL). Images were taken over 10 minutes covering approximately a 100° field. There were no adverse reactions to fluorescein. Images were dual graded by masked observers with adjudication, in The Liverpool Ophthalmic Reading Centre according to a standardized protocol that classifies the type and severity of FA features with good interrater reliability [23].

#### Brain Imaging and Grading

Images were acquired using a 0.35 T Signa Ovation Excite MRI scanner (General Electric, Milwaukee, WI) as reported previously [2]. Patients were comatose and not sedated. Two radiologists independently interpreted each MRI, masked to clinical outcome and retinopathy status. Differences were resolved by consensus according to prespecified criteria [24]. Brain volume was scored according to the appearance of the cerebral hemispheres on axial T2-weighted images using a scale from 1 to 8. Scores of 7 and 8 were prespecified as life-threatening brain swelling, and involved marked sulcal effacement, without (score 7) or with evidence of uncal, subfalcine, or tonsillar herniation (score 8) [2]. Agreement about the presence of severely increased brain volume is 87%, with kappa 0.73 (95% confidence interval [CI], 0.61–0.83).

### Retinal Histopathology

Single eyes from 21 subjects with retinopathy-positive CM included in the autopsy component of the research program (1996–2010) were analyzed as described previously [14]. We scored the histopathological severity of retinopathy according to a scale of intensity of retinal sequestration and maturation of sequestered parasites [14].

After fixation in 10% v/v buffered formalin, ocular specimens were opened horizontally in the pupil-optic nerve plane or by an equatorial incision. Gross pathology assessment was performed with a dissecting microscope, and retinopathy features were photographed. All samples were dehydrated and embedded in paraffin wax before 4-μm thick sections were cut with a manual rotary microtome (at least 100 sections per specimen). We investigated white-centered hemorrhages by making serial sections on 42 hemorrhages from 15 cases. Four hemorrhages were isolated by punch biopsy and embedded separately. Sequential sections were stained with hematoxylin and eosin (for staging and hemozoin), Martius-Scarlet-Blue (for fibrin), periodic acid-Schiff (for platelet-fibrin clots), and by immunohistochemistry to assess vessel integrity, clotting, and inflammation (Supplementary Table 1). We examined a minimum of 50 capillaries and 50 venules (diameter of 5–50 µm) per case using an Olympus BX60 system microscope.

### Statistical Analysis

We assessed individual variables graphically and numerically and collapsed categories if less than 5 to ensure stable estimation. We collapsed the original brain swelling variable: combining grades 1–3, 4–6, and 7–8 (severe). We used data from 1 eye per patient (more severely affected for each variable) and excluded subjects with missing data. We compared eligible patients who had admission imaging with those who did not to assess selection bias (Supplementary Tables 2 and 3).

We used a multiple correspondence analysis (MCA) to explore associations between FA variables, clinical outcome, and severe brain swelling [25]. This analysis allows visualization of multivariate associations in 2 dimensions and does not test the statistical significance of individual associations. To do this, we analyzed (1) clinical outcome as a nominal dependent variable (with multinomial logistic regression) and (2) brain swelling as an ordinal dependent variable (ordered logistic regression). We conducted a mediation analysis to clarify the relationship between retinal leak, severe brain swelling, and death [26, 27].

We tested associations with histological features using analysis of variance (ANOVA) and Spearman rank correlation. We report odds ratios (ORs) with 95% CIs and considered the 5% level to be significant. We used Stata, version 13 (StataCorp, College Station, TX).

## RESULTS

### Fluorescein Angiography

We performed FA on 260 children with CM of 549 admissions with malarial retinopathy, and 134 also had brain MRI (Supplementary Figure 1 shows the cohort derivation). Fluorescein angiography was completed on the day of admission in 90% and MRI in 80%. All completed imaging within 48 hours.

The group’s characteristics are summarized in Supplementary Tables 2 and 3. Subjects having FA on average had worse malarial retinopathy on funduscopy, longer coma, more convulsions, lower lactate, higher LP opening pressure, and more neurological sequelae than patients who did not (*P* < .05). Subjects who had FA and MRI had a longer median coma duration, more neurological sequelae, lower lactate, and worse malarial retinopathy than patients who had neither (*P* < .05).

**Figure 1.**
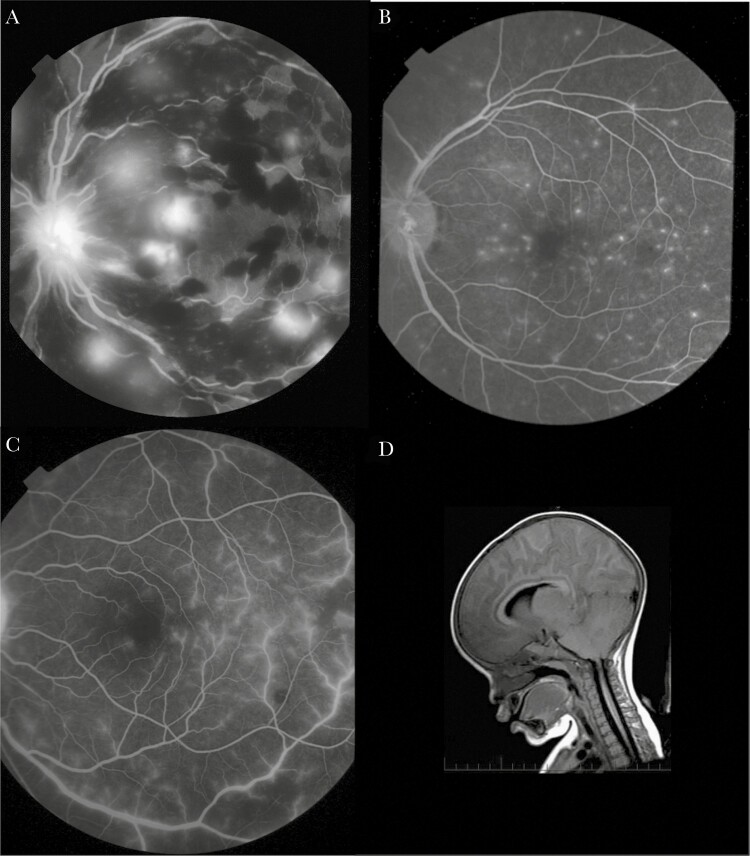
Retinal leakage and severe brain swelling seen in pediatric cerebral malaria. (A) Fluorescein angiographic image showing multiple large focal leaks (LFL). An LFL is a large leak of fluorescein from a vessel within the retina. Note associated black masking from recent multiple blot retinal hemorrhages. Clusters of more established hemorrhages around the vascular arcades show a white central dot of the fibrin core. The optic disc has abnormal fluorescein leakage from disc swelling (papilloedema). (B) Fluorescein angiographic image showing many punctate leaks (PL). A PL is a small fluorescein leak from deep retina or underlying retinal pigment epithelium. (C) Fluorescein angiographic image showing widespread leakage from larger venules and postcapillary venules (vessel leak). (D) Sagittal magnatic resonance imaging of the brain showing severe brain swelling in a child with retinopathy-positive cerebral malaria, with herniation of the cerebellum at the foramen magnum (arrow).

Three patterns of fluorescein leakage were identified: large focal leak (LFL), punctate leak (PL), and vessel leak (Figure 1). Vessel leak was predominantly postcapillary venule leak and larger venule leak, which were analyzed separately. Vessel leak could be widespread or affecting short segments. Capillary nonperfusion (CNP), in the macula and fundus periphery, was seen in almost all subjects (the frequencies of FA features are shown in Table 1).

**Table 1.** Frequency of Fluorescein Angiogram Signs, Brain Swelling on MRI, and Clinical Outcomes^a^

Variable		Subjects With Admission FA and MRI n = 134 Recruited 2009 to 2014		Subjects With Admission FA n = 260 Recruited 2006 to 2014	
	Severity Grade	%	Number	%	Number
Macular CNP	Grade 0 or 1	6.87	131	12.16	255
	Grade 2	47.33		45.9	
	Grade 3 or 4	45.80		41.96	
Peripheral CNP	Grade 0 or 1	39.85	133	42.8	259
	Grade 2	24.81		24.32	
	Grade 3 or 4	35.34		32.8	
Punctate leak	None	63.43	134	67.7	260
	1–5 sites	28.36		26.9	
	>5 sites	8.21		5.1	
Large focal leak	None	83.58	134	81.9	260
	1 site	6.72		8.1	
	>1 site	9.70		10.0	
Larger Venule leak	None	56.39	133	56.59	258
	Grade 1	32.33		28.68	
	Grade 2 or 3	11.28		14.73	
Postcapillary venule leak	None or grade 1	75.19	133	70.8	257
	Grade 2	17.29		19.84	
	Grade 3 or 4	7.52		9.3	
Optic disc leak	Absent	18.66	134	13.85	260
	Present	81.34		86.15	
IVFD in large arterioles	Absent	86.15	130	84.74	249
	Present	13.85		15.26	
Clinical outcome	Full recovery	73.9	134	74.6	260
	Sequelae	11.9		11.9	
	Death	14.2		13.5	
Brain swelling	Grade 1–3	13.5	133	n/a	
	Grade 4	28.6		n/a	
	Grade 5	20.3		n/a	
	Grade 6	21.8		n/a	
	Grade 7 or 8	15.8		n/a	

Abbreviations: CNP, capillary nonperfusion; FA, fluorescein angiography; IVFD, intravascular filling defect; MRI, magnatic resonance imaging; n/a, not applicable.

^a^Missing data are due to ungradable images.

### Associations Between Fluorescein Angiography Features and Outcome

**Figure 2.**
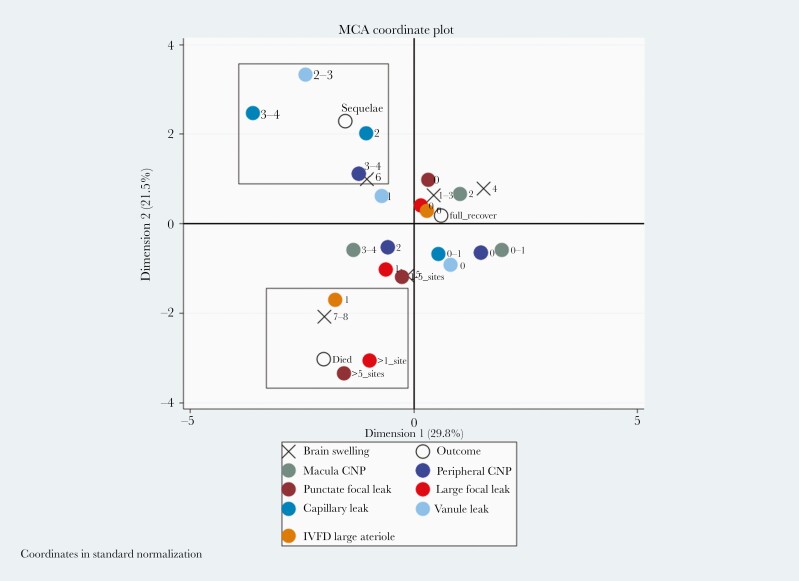
Multiple correspondence analysis (MCA) plot showing fluorescein angiogram features cluster with different outcomes in children with cerebral malaria. This analysis looks for multiple associations in 2 dimensions, and the boxes are illustrative. The severe grade of large focal leak (2), punctate leak (2), presence of arteriolar intravascular filling defects (IVDF) (1), and severe brain swelling (grades 7–8) cluster with death. More severe grades of larger venule (2) and postcapillary venule (2–3) leak and capillary nonperfusion (CNP) in the retinal periphery (3) cluster with neurological sequelae. Absent or mild angiographic features cluster with full recovery on discharge. Disc leak and large venule IVFD, which were plotted close to the origin, have been omitted from the plot for clarity. Zero indicates the absence of a feature and ascending numbers indicate worsening severity.

An MCA of FA features, severe brain swelling, and outcome shows separate clusters around recovery, death, and neurological sequelae (Figure 2). Both LFL and PL cluster with death and severe brain swelling. Vessel leak and peripheral CNP cluster with neurological sequelae. These associations were controlled for all plotted variables.

The MCA findings were confirmed with regression models of clinical outcome. Multinomial logistic regression revealed unadjusted associations between death and LFL (>1 site, 13.20, 5.21–33.78) and PL (>5 sites 8.58, 2.56–29.08), (*P* < .001 for both). Neurological sequelae were associated with postcapillary venule leak (grade 3–4, 3.71, 1.26–11.02) (*P* = .02), and peripheral CNP (grade 3–4, 2.69, 1.07–6.83) (*P* = .04) (Table 2). Macular CNP grade 4 was associated with neurological sequelae (9.06, 1.79–45.90) (*P* = .008) and death (11.52, 1.30–102.02) (*P* = .03).

**Table 2.** Unadjusted Associations Between Retinal Angiographic Features and Outcomes (Recovery With Neurological Sequelae, or Death) With Reference to Subjects Who Recovered Fully^a^

FA Feature	Outcome	FA Grade	Odds Ratio	*P*	95% Confidence Interval	N
Punctate leak	Sequelae	1–5 sites	0.55	.25	0.2 to 1.52	260
		>5 sites	0.00	.98	0.00 to >1000	
	Death	1–5 sites	4.06	**<.001**	1.82 to 9.12	
		>5 sites	8.58	**<.001**	2.56 to 29.08	
Large focal leak	Sequelae	1 site	1.62	.42	0.50 to 5.21	260
		>1 site	0.64	.68	0.08 to 5.26	
	Death	1 site	0.55	.58	0.07 to 4.39	
		>1 site	13.20	**<.001**	5.21 to 33.78	
Postcapillary venule leak	Sequelae	Grade 2	1.70	.26	0.67 to 4.26	257
		Grade 3–4	3.71	**.02**	1.26 to 11.02	
	Death	Grade 2	0.25	.07	0.06 to 1.11	
		Grade 3–4	1.48	.52	0.45 to 4.81	
Larger venule leak	Sequelae	Grade 1	1.86	.16	0.78 to 4.39	258
		Grade 2–3	2.51	.08	0.90 to 6.89	
	Death	Grade 1	1.28	.56	0.55 to 3.00	
		Grade 2–3	1.63	.35	0.59 to 4.57	
Macular capillary nonperfusion	Sequelae	Grade 2	1.59	.56	0.33 to 7.59	255
		Grade 3	1.92	.43	0.37 to 9.88	
		Grade 4	9.06	**.008**	1.79 to 45.90	
	Death	Grade 2	2.60	.38	0.32 to 21.39	
		Grade 3	7.69	.06	0.96 to 61.55	
		Grade 4	11.52	**.03**	1.30 to 102.02	
Peripheral capillary nonperfusion	Sequelae	Grade 2	2.32	.10	0.84 to 6.44	259
		Grade 3–4	2.69	.04	1.07 to 6.83	
	Death	Grade 2	1.72	.24	0.69 to 4.29	
		Grade 3–4	1.54	.33	0.65 to 3.66	

Abbreviations: FA, fluorescein angiography.

^a^Associations were estimated using multinomial logistic regression, in 260 subjects with admission fluorescein angiogram. The reference category is absence of a feature (except capillary leak, macular capillary nonperfusion, and peripheral capillary nonperfusion where Grades 0 and 1 were combined due to small numbers without these features). The odds ratio estimate is equal to exponential of the coefficient. *P*≤ .05 are in bold.

### Associations Between Fluorescein Angiography Features and Severe Brain Swelling

Ordered logistic regression of FA features and brain swelling revealed significant unadjusted associations with PL (>5 sites: 3.6, 1.2–11.1, *P* = .02) and LFL (>1 site: 4.8, 1.5 to 15.5, *P* = .01) (Table 3) but not with vessel leak or CNP.

**Table 3.** Unadjusted Associations Between Angiography Features and Brain Swelling^a^

FA Feature	FA Grade	Odds Ratio	*P*	95% CI	n
Punctate leak	1–5 sites	0.78	.47	0.39 to 1.54	133
	>5 sites	3.62	**.02**	1.19 to 11.09	
Large focal leak	1 site	0.47	.25	0.13 to 1.69	133
	>1 site	4.77	**.01**	1.47 to 15.46	
Postcapillary venule leak	Grade 2	0.76	.50	0.35 to 1.68	132
	Grade 3–4	2.94	.09	0.83 to 10.36	
Larger venule leak	Grade 1	1.09	.80	0.56 to 2.15	132
	Grade 2–3	1.85	.24	0.67 to 5.14	
Macular capillary nonperfusion	Grade 2	0.84	.79	0.22 to 3.01	130
	Grade 3	1.97	.32	0.52 to 7.41	
	Grade 4	1.94	.36	0.47 to 7.98	
Peripheral capillary nonperfusion	Grade 1	3.06	.47	0.15 to 63.30	132
	Grade 2	3.07	.47	0.14 to 64.70	
	Grade 3	2.67	.53	0.12 to 58.27	
	Grade 4	2.97	.49	0.14 to 63.67	

Abbreviations: CI, confidence interval; FA, fluorescein angiography.

^a^The sample is 134 subjects with both admission fluorescein angiogram and magnatic resonance imaging brain. Associations were estimated using ordered logistic regression, *P* < .05 in bold.

Mediation analysis showed that the association between LFL and death was consistent with mediation via brain swelling rather than directly, assuming no significant confounders (*P* = .02) (Supplementary Table 4). The association of PL to death was not significant as a binary variable due to smaller numbers with severe PL (>5 sites).

### Pathogenesis of Fluorescein Angiography Features

**Figure 3.**
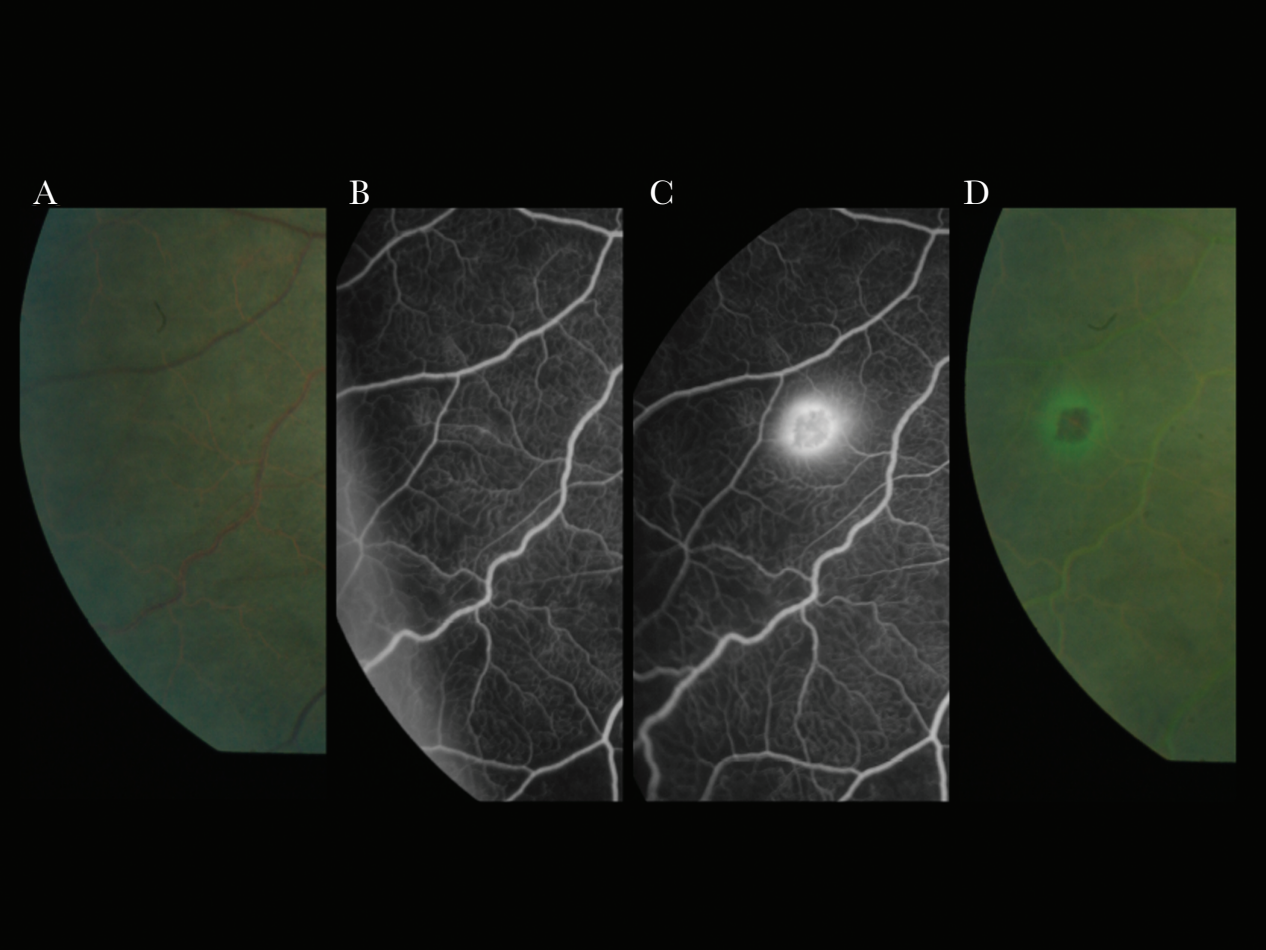
Development of large focal leak and colocated retinal hemorrhage during angiogram. From left to right: (A) preangiogram color image; (B) fluorescein angiogram at 6 minutes; (C) fluorescein angiogram at 9 minutes, large focal leak has developed; and (D) color image immediately postangiogram shows a hemorrhage at the same site, with a halo of fluorescein.

Serial images from 6 subjects demonstrated progression from LFL into a new blot or white-centered hemorrhage (Figure 3). In contrast, PL did not correlate with obvious features on color images. Vessel leak was common and sometimes seen adjacent to areas of CNP, which may be reperfusing (Supplementary Figure 2). Breakdown of the BRB or vessel leak in ischemic zones is a feature of other retinal conditions such as diabetic retinopathy [28].

### Histopathology

Single eyes from 21 subjects were available for histopathology, 4 of whom had FAs in life.

#### Retinal White-Centered Hemorrhages and Large Focal Leak

**Figure 4.**
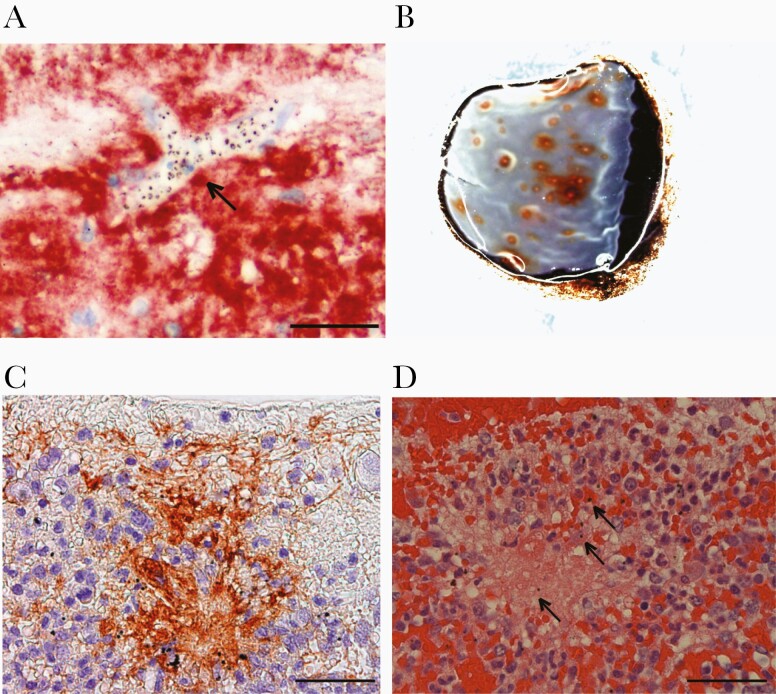
Histopathology of vascular leakage and white-centered hemorrhages. (A) Fibrinogen around a heavily parasitized microvessel (arrow), shown in red by immunohistochemistry, with blue hematoxylin counterstaining. Scale bar = 100 µm. (B) Gross pathology of a superior calotte used to directly sample white-centered hemorrhages in a case with moderate to severe malarial retinopathy. (C) White-centered hemorrhage has a core of fibrinogen (immunohistochemistry). Scale bar = 100 µm. (D) Fibrinogen confirmed by hematoxylin and eosin. Hemozoin is visible as dark brown dots (arrows). Scale bar = 50 µm.

We analyzed sections from 15 cases with white-centered hemorrhage (42 hemorrhages) including 3 with LFL. White-centered hemorrhages typically occurred in the deep capillary plexus and had a dense core of fibrinogen and fibrin (39 of 42 hemorrhages with fibrinogen [Figure 4]; 28 of 42 with fibrin). Vessel remnants were not commonly seen, suggesting the originating vessel was small or completely disrupted. Intact pRBCs were uncommon within hemorrhages, but hemozoin from ruptured parasitized erythrocytes was prominent and often internalized by phagocytes in situ. Platelets were relatively uncommon (CD61 positive, 12 of 42 hemorrhages). Leukocytes were abundant (CD45 positive, 39 of 42); predominantly morphological monocytes with cytoplasmic hemozoin (Supplementary Figure 3). Monocytes with hemozoin were also found in parasitized capillaries and venules without hemorrhage (median 30% [range, 16% to 64%], 50 vessels/eye from 21 eyes).

#### Vessel Leak

To investigate the characteristics of retinal vessel leakage, we looked for extravascular fibrinogen in relation to sequestration in all 21 cases. Extravascular fibrinogen was common in the perivascular space of retinal capillaries and venules in association with more severe retinopathy (*P* < .005, ANOVA) and associated with density of sequestration (Spearman rho = 0.56, *P* < .001) (Supplementary Figure 3). Fibrinogen was not visible around choroidal vessels, which have little or no sequestration [14].

#### Punctate Leak

Only 1 case with PL was available at autopsy, and they also had vessel leak on FA. Punctate leak was not evident funduscopically or on gross pathological examination. We were unable to determine any specific histopathological features attributable to PL.

## DISCUSSION

Sequestration and brain swelling are considered central pathological processes in CM [2, 29]. Brain swelling can result from vasogenic edema through BBB dysfunction [13], cytotoxic edema from hypoxia [30], and also hemorrhagic breaches in the BBB. We studied the retinal circulation to infer the contribution of these mechanisms to severe brain swelling, death, and neurodisability in CM. We show that BRB leakage is not homogeneous but composed of distinct types with different clinical associations. Although the types can coexist, LFL and PL associate with severe brain swelling and death, whereas vessel leakage and CNP associate with gross neurological sequelae. Our findings suggest that neurological sequelae and death are discrete categories, rather than part of the same scale of severity.

Large focal leak appears to indicate a new retinal hemorrhage. We observed the onset of LFL at sites where hemorrhages occurred over 10-minute angiograms (Figure 3). The number of retinal hemorrhages and a large increase in retinal hemorrhages are associated with death in CM [31] and correlate with cerebral ring hemorrhages [16]. Capturing 2 or more hemorrhages in formation during a 10-minute angiogram with a limited field of view indicates rapid accumulation of hemorrhages and LFL sites. We propose that LFL is a manifestation of rapid hemorrhage accumulation occurring in the CNS, indicating multiple focal BBB ruptures in the brain. These results suggest that multiple cerebral ring hemorrhages are a driver of fatal brain swelling through physical breaches of the BBB. The association between LFL (hemorrhage accumulation) and death is mediated by brain swelling, giving statistical support to the biological plausibility of this hypothesis. Hemorrhages in the retina and the brain are not just signs of collateral damage to capillaries and venules, but this investigation suggests they are an integral step in the development of severe brain swelling because any egress of blood cells will be accompanied by a significant fluid volume. Accumulating enough breaches in the BBB over a short time can overwhelm compensatory mechanisms and prove to be fatal.

Retinal white-centered hemorrhages have a core of fibrin(ogen) and monocytes with phagocytosed hemozoin. These are also evident in some small vessels without hemorrhage. These novel histological findings are consistent with the histopathology of cerebral ring hemorrhages [29] and presence of monocytes in brain vessels [32]. Monocytes are stimulated to release a prephagocytic oxidative burst by the combination of hemozoin and fibrinogen [33]. Occlusion of vessels with sequestered pRBCs alone does not cause hemorrhage [15]. Parasite binding to EPCR and consequent blockade of activated protein C (APC) may contribute to a proinflammatory as well as prothrombin state with unregulated thrombin generation and fibrin deposition [29, 34]. Hemorrhages and LFL could be related to interactions between schizont rupture, release of hemozoin and histidine rich protein 2 (HRP2), prothrombogenic state, and inflammatory responses from circulating monocytes. One or more of these processes result in rupture of capillary walls. Multiplied up countless times by synchronous schizont rupture, this could result in a rapid egress of fluid into the extracellular space within the cranial cavity.

Punctate leak is more difficult to characterize because it does not correspond to other FA or clinical features. We have not been able to identify a histological correlate. Even with stereoscopic images, it is unclear whether it arises from retinal capillaries or the retinal pigment epithelium that constitutes an outer BRB [23]. It was seen at 1–5 sites in approximately 25% of FAs, and >5 sites in only 5%, which is the group clustering with death in the MCA. Given the widespread and visible presence of sequestration in retinal vessels, it seems unlikely that PL is directly related to sequestration. Increased transluminal pressure could plausibly account for the appearance of PL, if sufficient to force fluorescein through the endothelium where integrity has been affected by loss of EPCR [29]. If such leakage also occurs in the brain, the association between PL and death might suggest that hydrostatic edema plays an important role in the 20% of autopsy cases without brain hemorrhage, where fibrinogen is seen around vessels packed with pRBCs [30]. Alternatively, PL may indicate failure of the outer BRB, a sign of severe systemic infection and tissue dysregulation, and when widespread, a preagonal event.

Vessel leak occurred in approximately 50% of CM patients with retinopathy on admission, and with CNP it was associated with the development of neurological sequelae. In admission FAs, vessel leak was often observed with CNP, and in subsequent FAs, it developed in vessels crossing, adjacent, or reperfusing areas of CNP (Supplementary Figure 3). Vessel leak is indicative of vasogenic edema, presumably mediated by endothelial activation from parasite factors (eg, HRP2) [35] and host factors (eg, angiopoetin-2) [29]. In the retina, it also occurs in concert with patchy tissue hypoxia and consequent cytotoxic edema. However, vessel leak is associated with neurological sequelae rather than severe brain swelling and death. This association may be mediated through CNP and associated reperfusion injury, with patchy CNP in the brain leading to neurological sequelae and more subtle neurocognitive deficits that commonly develop after CM [5] (but not tested here). Diffuse axonal injury and myelin damage is seen at autopsy associated with sequestration, but not ring hemorrhages, and is likely an effect of hypoxia [30]. Vasogenic oedema from endothelial barrier disruption [29] and vessel leak is insufficient to be associated with severe brain swelling, but the associated tissue hypoxia may cause deleterious effects on brain function in survivors.

Our results introduce the concept that neurological vessel leak (vasogenic edema) and CNP (ischemia) are typical states for CM, and survivable with a risk of neurological sequelae, but that severe brain swelling and death become much more likely in the face of multiple hemorrhagic breaks in the BRB/BBB represented by LFL and ring hemorrhages. In this model fatal brain swelling results from hemorrhagic leaks or physical breaches rather than diffuse disruption of endothelial tight junctions. Thus, death is not on a continuum of severity with neurological sequelae, but it is a distinct pathological process, and both may need separate mitigating interventions.

Our study has limitations. Selection bias is possible. Children with both very mild and very severe disease were less likely to tolerate retinal or brain imaging, and this is consistent with differences in retinopathy severity and coma duration in these groups. Missing mild and severe cases could lead to bias, and our results may not be generalizable to all degrees of disease severity. We present extensive descriptive data to allow comparisons with our study groups. Unmeasured confounders are possible in observational studies, although these were well characterized clinical cases. Another limitation is the lack of comparative brain histopathology from paired cases, because autopsies were discontinued after the introduction of FA.

## CONCLUSIONS

We studied the BRB to make inferences about brain and BBB pathology. In summary, our FA data show that leakage in the retina is not homogenous but consists of 3 types, which are associated with different clinical outcomes. This indicates that the events causing death versus neurological disability may be qualitatively distinct, rather than varying only by severity. Vessel leak is commonly seen in CM in relation to sequestration and the occlusion of capillaries and venules. It is associated with neurological sequelae, and we postulate that this is mediated in the brain by patchy hypoxic injury, akin to the features seen in the retina. By contrast, fatal outcome is associated with LFL caused by hemorrhage formation and a physical break in the BRB. The association between LFL and death is consistent with equivalent cerebral hemorrhage causing fatal brain swelling. Hemorrhagic breaches in the BBB with fluid egress, multiplied many times in the brain by schizont rupture and local coagulopathy, may cause fatal brain swelling and death. Punctate leak is more obscure but may relate to dysfunction of the outer BRB during a terminal or preagonal phase. Our data suggest that new treatments for CM need to target different mechanisms to reduce mortality and, on the other hand, to improve outcomes for survivors.

## Supplementary Data

Supplementary materials are available at The *Journal of Infectious Diseases* online. Consisting of data provided by the authors to benefit the reader, the posted materials are not copyedited and are the sole responsibility of the authors, so questions or comments should be addressed to the corresponding author.

**Supplementary Figure 1.** Derivation of cohorts. (A) Entire fluorescein angiogram cohort. (B) Fluorescein angiogram and MRI brain subset cohort. Ret- malarial retinopathy absent; Ret+ malarial retinopathy present.

**Supplementary Figure 2.** Capillary nonperfusion and leakage. (A) Admission color and fluorescein angiogram (FA) images with retinal whitening and peripheral capillary nonperfusion (CNP) and no leakage. Note the attenuated venule in center is orange, with intravascular filling defects on FA. (B) Day 1 showing development of larger zones of CNP and fluorescein leakage from vessels crossing or adjacent to nonperfused zones. (C) Day 2 showing improvement of CNP (reperfusion) and leakage from reperfusing vessels.

**Supplementary Figure 3.** Histopathology of monocytes and hemozoin. (A) Hemozoin-laden monocytes identified in the core of white-centered hemorrhages by the presence of dark brown malaria pigment and typical kidney-shaped nuclei. Cells are marked by arrows. Anti-CD45 immunohistochemistry (red) and hematoxylin (blue) counterstaining are shown. Scale bar = 20 µm. (B–D) Characterization of monocytes and hemozoin by hematoxylin and eosin staining. (B) Monocytes with phagocytosed hemozoin in capillaries. Scale bar = 10 µm. (C) Monocytes in venules. Scale bar = 20 µm. (D) Extra-erythrocytic hemozoin in retinal capillaries. Scale bar = 10 µm.

jiaa541_suppl_Supplementary_Figure_1

jiaa541_suppl_Supplementary_Figure_2

jiaa541_suppl_Supplementary_Figure_3

jiaa541_suppl_Supplementary_Table_1

jiaa541_suppl_Supplementary_Table_2

jiaa541_suppl_Supplementary_Table_3

jiaa541_suppl_Supplementary_Table_4

jiaa541_suppl_Supplementary_Figure_Legends

## Notes

***Acknowledgments.*** We are grateful to the patients and their guardians for taking part in the study, as well as the clinical and other staff on the pediatric research ward. We are grateful to Lucy Culshaw, Simon Biddolph, Steve Kamiza, and staff involved in performing autopsies. We thank Cowles Chilingulo and other magnatic resonance imaging (MRI) staff for performing MRIs. In addition to the authors, Simon Glover also performed retinal examinations and ocular imaging and we are grateful for his contribution.

***Author contributions.*** N. A. V. B. had full access to all the data in the study and takes final responsibility for the decision to submit the manuscript for publication.

***Financial support.*** This work was funded by The Wellcome Trust (Grant Number 092668/Z/10/Z), *Malawi-Liverpool-Wellcome Trust Clinical Research Programme* (Core Grant Number 084679/Z/08/Z), and the *National Institutes of Health* (Grant Number 5R01AI034969).

***Potential conflicts of interest.*** All authors: No reported conflicts of interest. All authors have submitted the ICMJE Form for Disclosure of Potential Conflicts of Interest.

Presented in part: Association for Eye Research in Ophthalmology Annual Congress, 8 May 2017.
