# Supplementary figures and images for: How Does Blood-Retinal Barrier Breakdown Relate to Death and Disability in Pediatric Cerebral Malaria?
Source: J Infect Dis. 2020 Aug 26;225(6):1070–80. doi: 10.1093/infdis/jiaa541 (PMC8922008; doi:10.1093/infdis/jiaa541)

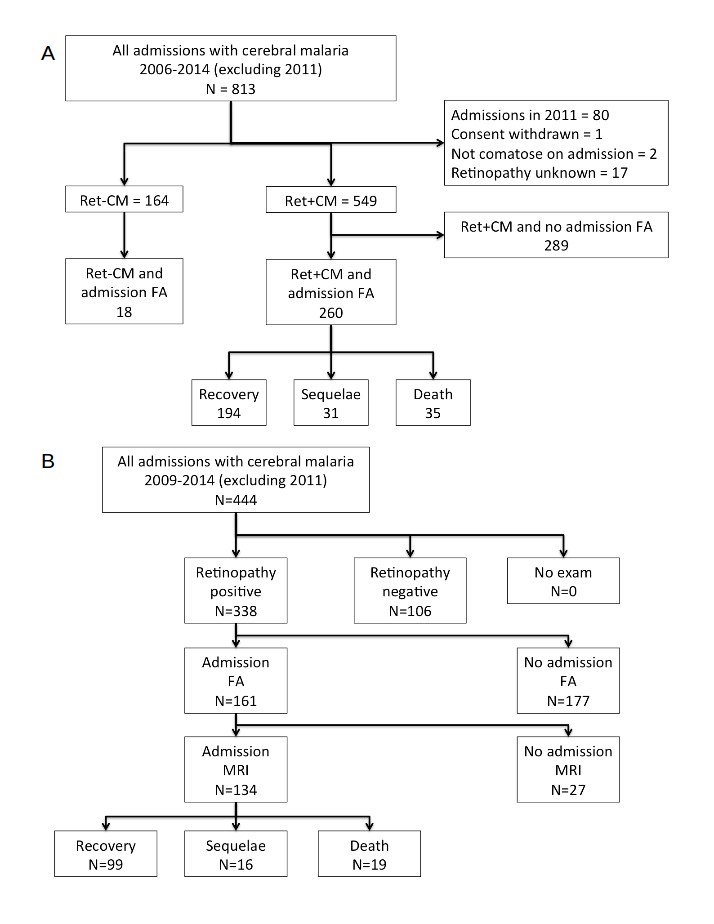

Supplement: jiaa541_suppl_Supplementary_Figure_1 [file jiaa541_suppl_supplementary_figure_1.jpeg]

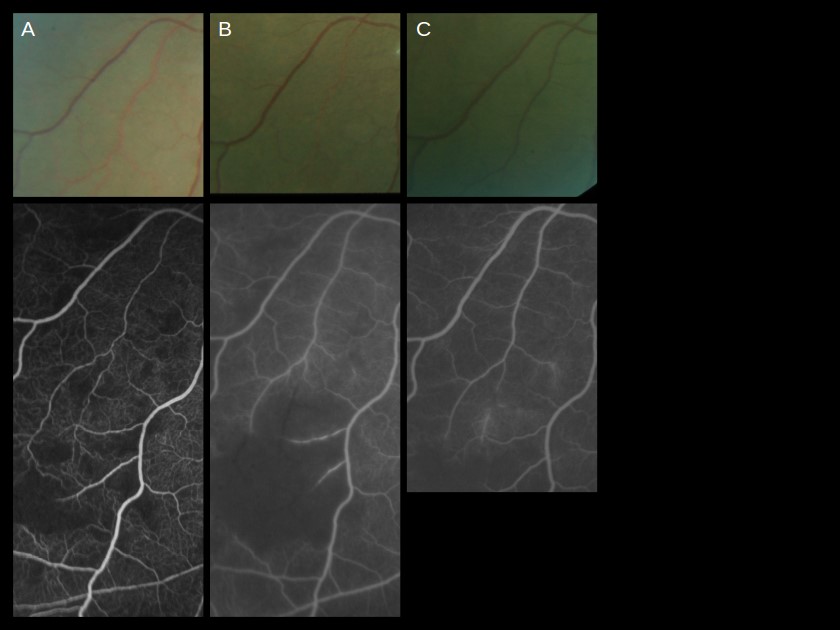

Supplement: jiaa541_suppl_Supplementary_Figure_2 [file jiaa541_suppl_supplementary_figure_2.jpeg]

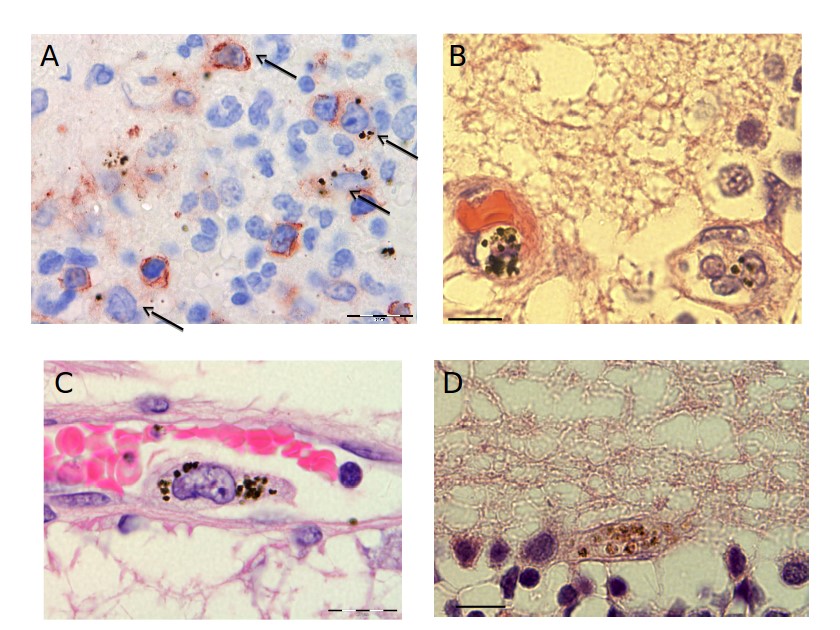

Supplement: jiaa541_suppl_Supplementary_Figure_3 [file jiaa541_suppl_supplementary_figure_3.jpeg]
