# Supplementary material for: How Does Blood-Retinal Barrier Breakdown Relate to Death and Disability in Pediatric Cerebral Malaria?
Source: J Infect Dis. 2020 Aug 26;225(6):1070–80. doi: 10.1093/infdis/jiaa541 (PMC8922008; doi:10.1093/infdis/jiaa541)
Supplement: jiaa541_suppl_Supplementary_Table_1 [file jiaa541_suppl_supplementary_table_1.docx]

**Supplementary Table 1**. Antigens used to characterize features of retinal leakage.

| **Antigen** | **Specificity** | **Feature** | **Manufacturer (clone)** | **Host* (class)** | **Ag retrieval †** | **Dilution ‡** | **Chromogen §** |
| --- | --- | --- | --- | --- | --- | --- | --- |
| CD34 (II) | Endothelium | Vessel integrity | Dako (QBEnd-10) | Ms mAb (IgG1k) | Heat (High pH) | 1:100, 30 min RT | DAB |
| Smooth muscle actin (SMA) | Pericyte | Vessel integrity | Dako (1A4) | Ms mAb (IgG2ak) | Heat (Low pH) | 1:2,000, o.n. 4°C | AEC |
| Laminin | Basal membrane | Vessel integrity | Sigma | Rb pAb | Proteinase K | 1:500, o.n. 4°C | AEC |
| Collagen IV | Basal membrane | Vessel integrity | Sigma (COL-94) | Ms mAb (IgG1) | Proteinase K | 1:2,000, o.n. 4°C | AEC |
| Fibrinogen | Plasma protein | Vessel integrity | Dako | Rb pAb | Proteinase K | 1:500, 30 min RT | AEC |
| Fibrin | Fibrin polymer | Clotting | 102-10 (gift from Dr Y Matsumura) | mAb-HRP conjugated | Heat (Low pH) | 1:100, o.n. 4°C | DAB |
| CD61 | Platelets and precursors | Clotting | Thermo Scientific | Ms mAb (IgG1) | Heat (High pH) | 1:100, 32 min RT | DAB or AEC |
| CD45 | Pan-leukocyte | Inflammation | Dako (2B11+PD7/26) | Ms mAb (IgG1k) | Heat (Low pH) | 1:200, o.n. 4°C | AEC |
| CD68 | Differentiated macrophages | Inflammation | Dako  (PG-M1) | Ms mAb (IgG3k) | Heat (Low pH) | 1:100, o.n. 4°C | AEC |

Host: Rb=rabbit; Ms=mouse; mAb=monoclonal antibody; pAb=polyclonal antibody. † Ag retrieval: heat-mediated antigen retrieval was performed in high pH solution (10mM Tris/1mM EDTA, pH 9.0) or low pH solution (trisodium citrate 10mM, pH 6.0). Proteinase K was from Dako (ready-to-use solution). ‡ Dilution and incubation time: RT=room temperature; o.n.=overnight. § Chromogen: AEC: 3-amino-9-ethylcarbazole; DAB=3,3'-diaminobenzidine.
