# Supplementary material for: How Does Blood-Retinal Barrier Breakdown Relate to Death and Disability in Pediatric Cerebral Malaria?
Source: J Infect Dis. 2020 Aug 26;225(6):1070–80. doi: 10.1093/infdis/jiaa541 (PMC8922008; doi:10.1093/infdis/jiaa541)
Supplement: jiaa541_suppl_Supplementary_Table_2 [file jiaa541_suppl_supplementary_table_2.docx]

**Supplementary Table 2**. Characteristics of eligible subjects comparing those not included (without admission FA) to those included (with admission FA) in the study. Retinal data are from the worst affected eye. All associations were estimated using logistic regression. P <=0.05 are in bold. CM = cerebral malaria, SMA = severe malarial anemia, DA = Disc area.

| **Variable** | **Detail** | **Subjects without admission FA** | | | | **Subjects with admission FA** | | | | **Association** | | |
| --- | --- | --- | --- | --- | --- | --- | --- | --- | --- | --- | --- | --- |
|  | | Median | IQR | % | number | Median | IQR | % | number | OR | 95%CI | p |
| Number | | n/a | n/a | n/a | 289 | n/a | n/a | n/a | 260 |  |  |  |
| **Demographic** | | | | | | | | | | | | |
| Age | months | 39 | 27-57.5 |  | 289 | 38.5 | 28-56 |  | 260 | 1 | 0.99-1.01 | 0.68 |
| Weight | kg | 12.2 | 10.1-14.95 |  | 289 | 12 | 10-15 |  | 260 | 0.99 | 0.96-1.03 | 0.73 |
| Height | cm | 92 | 84-103 |  | 283 | 92.5 | 84-104 |  | 256 | 0.99 | 0.99-1.01 | 0.87 |
| Sex | male |  | 146 | 50.52 | 289 |  | 126 | 48.46 | 260 | 1.09 | 0.78-1.52 | 0.63 |
|  | female |  | 143 | 49.48 |  |  | 134 | 51.54 |  |  |  |  |
| **Clinical** | | | | | | | | | | | | |
| Duration of fever pre-admission | hours | 60 | 43.5-72 |  | 284 | 60 | 48-72 |  | 247 | 1 | 0.99-1.01 | 0.26 |
| Duration of coma pre-admission | hours | 7 | 4-12 |  | 227 | 9 | 5-22.75 |  | 200 | 1.01 | 0.99-1.02 | 0.12 |
| Rectal temperature | ºC | 39 | 38.05-39.6 |  | 289 | 38.8 | 38.1-39.57 |  | 260 | 0.97 | 0.85-1.12 | 0.7 |
| Pulse | Beat/min | 160 | 140-176 |  | 289 | 151 | 134-171 |  | 260 | 0.99 | 0.98-0.99 | **0.009** |
| Systolic blood pressure | mmHg | 95 | 87.5-104 |  | 277 | 97 | 89.25-105.75 |  | 244 | 1.01 | 0.99-1.02 | 0.077 |
| Respiratory rate | Breath/min | 48 | 40-56 |  | 287 | 42.5 | 36-52 |  | 260 | 0.98 | 0.97-0.99 | **0.008** |
| CSF opening pressure | mmCSF | 150 | 110-190 |  | 148 | 170 | 116.25-220 |  | 134 | 1 | 1.00-1.01 | **0.046** |
| Jaundice | negative |  | 228 | 92.31 | 247 |  | 218 | 92.37 | 236 | 0.99 | 0.51-1.94 | 0.98 |
|  | positive |  | 19 | 7.63 |  |  | 18 | 7.63 |  |  |  |  |
| Respiratory distress | negative |  | 178 | 61.59 | 289 |  | 175 | 67.31 | 260 | 0.78 | 0.55-1.11 | 0.16 |
|  | positive |  | 111 | 38.41 |  |  | 85 | 32.69 |  |  |  |  |
| Diagnosis | CM |  | 133 | 46.02 | 289 |  | 110 | 42.31 | 260 | 1.08 | 0.91-1.28 | 0.38 |
|  | CM+SMA |  | 156 | 53.98 |  |  | 150 | 57.69 |  |  |  |  |
| Blantyre Coma score | 0 |  | 29 | 10.03 | 289 |  | 18 | 6.92 | 260 |  |  |  |
|  | 1 |  | 122 | 42.21 |  |  | 120 | 46.15 |  | 1.58 | 0.84-3.00 | 0.158 |
|  | 2 |  | 138 | 47.75 |  |  | 122 | 46.92 |  | 1.42 | 0.75-2.69 | 0.276 |
| Hours to reach coma score 3 | < 12 hrs |  | 82 | 34.45 | 238 |  | 27 | 12.39 | 218 |  |  |  |
|  | 12 to 24 hrs |  | 82 | 34.45 |  |  | 82 | 37.61 |  | 3.04 | 1.78-5.17 | **<0.001** |
|  | > 24 hrs |  | 74 | 31.09 |  |  | 109 | 50 |  | 4.47 | 2.64-7.57 | **<0.001** |
| Clinical outcome | full recovery |  | 225 | 77.85 | 289 |  | 194 | 74.62 | 260 |  |  |  |
|  | sequelae |  | 13 | 4.5 |  |  | 31 | 11.92 |  | 2.77 | 1.41-5.43 | **0.003** |
|  | death |  | 51 | 17.65 |  |  | 35 | 13.46 |  | 0.8 | 0.5-1.27 | 0.342 |
| History of convulsions pre-admission | negative |  | 64 | 22.3 | 287 |  | 47 | 18.29 | 257 | 1.28 | 0.84-1.95 | 0.25 |
|  | positive |  | 223 | 77.7 |  |  | 210 | 81.71 |  |  |  |  |
| Witnessed convulsions on admission | negative |  | 251 | 87.46 | 287 |  | 222 | 86.38 | 257 | 1.1 | 0.67-1.81 | 0.71 |
|  | positive |  | 36 | 12.54 |  |  | 35 | 13.62 |  |  |  |  |
| Witnessed convulsions after admission | negative |  | 192 | 66.44 | 289 |  | 143 | 55 | 260 | 1.62 | 1.15-2.29 | **0.006** |
|  | positive |  | 97 | 33.56 |  |  | 117 | 45 |  |  |  |  |
| **Investigations** | | | | | | | | | | | | |
| Peripheral parasitemia | cells | 72807.5 | 15792-301000 |  | 280 | 47720 | 3295.5-210000 |  | 252 | 0.99 | 0.99-1.00 | 0.19 |
| White cell count | cells | 9950 | 6800-15100 |  | 274 | 10000 | 7200-14400 |  | 247 | 1 | 0.99-1.00 | 0.84 |
| Platelet count | platelets | 54000 | 31000-84000 |  | 275 | 59000 | 31000-103000 |  | 245 | 1 | 0.99-1.00 | 0.14 |
| Hematocrit | % | 19.9 | 15.5-24.05 |  | 285 | 19.3 | 15.3-24.1 |  | 257 | 1.01 | 0.98-1.03 | 0.66 |
| Lactate | mmol/L | 6.9 | 3.8-11.6 |  | 287 | 4.85 | 2.9-9.175 |  | 256 | 0.94 | 0.90-0.97 | **0.001** |
| HRP2 | ng/ml | 6765.5 | 2827-12203 |  | 280 | 7641 | 3275-10471 |  | 259 | 1 | 0.99-1.00 | 0.73 |
| HIV status | negative |  | 223 | 86.1 | 259 |  | 203 | 84.94 | 239 | 1.1 | 0.67-1.81 | 0.71 |
|  | positive |  | 36 | 13.9 |  |  | 36 | 15.06 |  |  |  |  |
| **Retinal** | | | | | | | | | | | | |
| Retinal hemorrhages | none |  | 76 | 28.46 | 267 |  | 60 | 23.17 | 259 |  |  |  |
|  | 1 to 5 |  | 104 | 38.95 |  |  | 92 | 35.52 |  | 1.12 | 0.72-1.74 | 0.612 |
|  | 6 to 20 |  | 53 | 19.85 |  |  | 52 | 20.08 |  | 1.24 | 0.75-2.07 | 0.404 |
|  | 21 to 50 |  | 19 | 7.12 |  |  | 23 | 8.88 |  | 1.53 | 0.76-3.07 | 0.228 |
|  | >50 |  | 15 | 5.62 |  |  | 32 | 12.36 |  | 2.7 | 1.34-5.44 | **0.005** |
| Papilledema | negative |  | 204 | 76.4 | 267 |  | 175 | 67.57 | 259 | 1.55 | 1.06-2.28 | **0.024** |
|  | positive |  | 63 | 23.6 |  |  | 84 | 32.42 |  |  |  |  |
| Disc hyperemia | negative |  | 183 | 69.58 | 263 |  | 177 | 69.96 | 253 | 0.98 | 0.67-1.43 | 0.925 |
|  | positive |  | 80 | 30.42 |  |  | 76 | 30.04 |  |  |  |  |
| Macular whitening | none |  | 29 | 11.07 | 262 |  | 21 | 8.14 | 258 |  |  |  |
|  | <1/3DA |  | 159 | 60.69 |  |  | 97 | 37.6 |  | 0.84 | 0.46-1.56 | 0.585 |
|  | 1/3-1DA |  | 49 | 18.7 |  |  | 78 | 30.23 |  | 2.2 | 1.13-4.28 | **0.02** |
|  | >1DA |  | 25 | 9.54 |  |  | 62 | 24.03 |  | 3.42 | 1.65-7.1 | **0.001** |
| Foveal whitening | none |  | 58 | 22.22 | 261 |  | 35 | 13.62 | 257 |  |  |  |
|  | <1/3 fovea |  | 150 | 57.47 |  |  | 111 | 43.19 |  | 1.23 | 0.75-2.0 | 0.411 |
|  | 1/3-2/3 fovea |  | 30 | 11.49 |  |  | 52 | 20.23 |  | 2.87 | 1.55-5.31 | **0.001** |
|  | >2/3 fovea |  | 23 | 8.81 |  |  | 59 | 22.96 |  | 4.25 | 2.24-8.05 | **<0.001** |
| Temporal whitening | none |  | 73 | 28.19 | 259 |  | 35 | 13.78 | 254 |  |  |  |
|  | grade 1 |  | 140 | 54.05 |  |  | 101 | 39.76 |  | 1.5 | 0.93-2.43 | 0.09 |
|  | grade 2 |  | 28 | 10.81 |  |  | 53 | 20.87 |  | 3.95 | 2.15-7.27 | **<0.001** |
|  | grade 3 |  | 18 | 6.95 |  |  | 65 | 25.59 |  | 7.53 | 3.89-14.56 | **<0.001** |
| Orange vessels temporal periphery | absent |  | 196 | 77.78 | 252 |  | 122 | 62.89 | 194 | 2.07 | 1.36-3.13 | **0.001** |
|  | present |  | 56 | 22.22 |  |  | 72 | 37.11 |  |  |  |  |
| White vessels, temporal periphery | absent |  | 179 | 71.03 | 252 |  | 148 | 76.29 | 194 | 0.76 | 0.5-1.17 | 0.214 |
|  | present |  | 73 | 28.97 |  |  | 46 | 23.71 |  |  |  |  |
| White capillaries, temporal periphery | absent |  | 154 | 61.11 | 252 |  | 143 | 73.71 | 194 | 0.56 | 0.37-0.84 | **0.005** |
|  | present |  | 98 | 38.89 |  |  | 51 | 26.29 |  |  |  |  |

Clinical data from the history, examination, and standard investigations were collected routinely at admission. These included rectal temperature, full blood count (Coulter Counter, Beckman Coulter), and HIV status using two separate tests (Uni-Gold Recombigen HIV-1/2, Trinity Biotech; and Determine HIV-1/2, Inverness Medical). Finger prick blood samples were analyzed to determine parasite species and density, packed-cell volume, blood glucose and blood lactate (Lactate Pro point of care detector (Arkay Inc)). Histidine rich protein 2 (HRP2) was measured retrospectively in stored plasma (Cellabs ELISA).
