# Supplementary material for: How Does Blood-Retinal Barrier Breakdown Relate to Death and Disability in Pediatric Cerebral Malaria?
Source: J Infect Dis. 2020 Aug 26;225(6):1070–80. doi: 10.1093/infdis/jiaa541 (PMC8922008; doi:10.1093/infdis/jiaa541)
Supplement: jiaa541_suppl_Supplementary_Table_3 [file jiaa541_suppl_supplementary_table_3.docx]

**Supplementary Table 3**. Characteristics of included subjects comparing those having admission MRI to those not having admission MRI. Retinal variables are from the worst affected eye. p-values with an * were generated from Kruskal-Wallis test. All other associations were estimated using logistic regression. P <=0.05 are in bold. CM = cerebral malaria, SMA = severe malarial anemia, DA = Disc area.

| **Variable** | **Detail** | **Subjects with FA and MRI** | | | | **Subjects with FA but without MRI** | | | | **Association** | | |
| --- | --- | --- | --- | --- | --- | --- | --- | --- | --- | --- | --- | --- |
|  |  | Median | IQR | % | number | Median | IQR | % | number | OR | 95%CI | p |
| Number | |  |  |  | 134 |  |  |  | 27 |  |  |  |
| **Demographic** | | | | | | | | | | | | |
| Age | months | 43 | 27-66 |  | 134 | 33 | 23-54 |  | 27 | 1.01 | 0.99-1.03 | 0.22 |
| Weight | kg | 11.9 | 10-15 |  | 134 | 11 | 9-15 |  | 27 | 1.03 | 0.94-1.14 | 0.51 |
| Height | cm | 93.0 | 81-106 |  | 132 | 93 | 81-104 |  | 26 | 1.01 | 0.98-1.04 | 0.49 |
| Sex | male |  |  | 51.5 | 134 |  |  | 44.4 | 27 | 0.75 | 0.33-1.73 | 0.51 |
|  | female |  |  | 48.5 |  |  |  | 55.6 |  |  |  |  |
| **Clinical** | | | | | | | | | | | | |
| Duration of fever pre-admission | hours | 64 | 48-72 |  | 128 | 48 | 42-72 |  | 26 | 1.00 | 0.99-1.02 | 0.52 |
| Duration of coma pre-admission | hours | 9 | 5-21 |  | 105 | 9 | 5-18 |  | 21 | 1.01 | 0.98-1.04 | 0.57 |
| Rectal temperature | ºC | 38.9 | 38.1-39.7 |  | 134 | 38.8 | 38.1-39.4 |  | 27 | 1.06 | 0.74-1.52 | 0.74 |
| Pulse | Beat/min | 149 | 132-169 |  | 134 | 152 | 130-171 |  | 27 | 1.00 | 0.98-1.02 | 0.97 |
| Systolic blood pressure | mmHg | 96 | 89-104 |  | 120 | 95 | 86-101 |  | 26 | 1.02 | 0.99-1.06 | 0.23 |
| Respiratory rate | Breath/min | 41 | 36-52 |  | 134 | 48 | 38-56 |  | 27 | 0.99 | 0.95-1.02 | 0.44 |
| CSF opening pressure | mmCSF | 170 | 130-232 |  | 62 | 185 | 122-217 |  | 16 | 1.00 | 0.99-1.01 | 0.92 |
| Jaundice | negative |  |  | 96.3 | 134 |  |  | 92.6 | 27 | 0.48 | 0.09-2.64 | 0.4 |
|  | positive |  |  | 3.7 |  |  |  | 7.4 |  |  |  |  |
| Respiratory distress | negative |  |  | 72.4 | 134 |  |  | 66.6 | 27 | 0.76 | 0.31-1.85 | 0.55 |
|  | positive |  |  | 27.6 |  |  |  | 33.3 |  |  |  |  |
| Diagnosis | CM |  |  | 42.5 | 134 |  |  | 51.8 | 27 | 1.21 | 0.8-1.83 | 0.37 |
|  | CM+SMA |  |  | 57.5 |  |  |  | 48.2 |  |  |  |  |
| Coma score | 0 |  |  | 6.7 | 134 |  |  | 7.4 | 27 |  |  |  |
|  | 1 |  |  | 51.5 |  |  |  | 33.3 |  | 1.7 | 0.32-9.16 | 0.53 |
|  | 2 |  |  | 41.8 |  |  |  | 59.3 |  | 0.78 | 0.15-3.97 | 0.76 |
| Time to reach coma score 3 | hours | 28 | 18-52 |  | 109 | 16 | 10-28 |  | 23 | 1.01 | 0.99-1.02 | **0.006*** |
| Clinical outcome | full recovery |  |  | 73.9 | 134 |  |  | 77.8 | 27 |  |  |  |
|  | sequelae |  |  | 11.9 |  |  |  | 3.7 |  | 3.39 | 0.43-27.0 | 0.25 |
|  | death |  |  | 14.2 |  |  |  | 18.5 |  | 0.81 | 0.27-2.40 | 0.69 |
| Witnessed convulsions on admission | negative |  |  | 17.9 | 134 |  |  | 19.2 | 26 |  |  |  |
|  | positive |  |  | 82.1 |  |  |  | 80.8 |  | 1.09 | 0.37-3.18 | 0.87 |
|  |  |  |  |  |  |  |  |  |  |  |  |  |
| Witnessed convulsions after admission | negative |  |  | 85.5 | 131 |  |  | 92.6 | 27 |  |  |  |
|  | positive |  |  | 14.5 |  |  |  | 7.4 |  | 2.12 | 0.46-9.70 | 0.33 |
| **Investigations** | | | | | | | | | | | | |
| Peripheral parasitemia | cells | 39360 | 1270-176000 |  | 129 | 50550 | 19200-182000 |  | 26 | 1.00 | 1.00-1.00 | 0.436 |
| White cell count | cells | 10200 | 6500-14850 |  | 125 | 9500 | 7650-12950 |  | 26 | 1.00 | 1.00-1.00 | 0.63 |
| Platelet count | platelets | 58000 | 30000-97000 |  | 123 | 47500 | 25750-86750 |  | 26 | 1.00 | 1.00-1.00 | 0.68 |
| Hematocrit | % | 20 | 17-25.1 |  | 131 | 18.7 | 15.6-23.2 |  | 27 | 1.05 | 0.98-1.12 | 0.18 |
| Lactate | mmol/L | 4.6 | 2.8-8.95 |  | 134 | 4.6 | 3.0-11.3 |  | 23 | 0.96 | 0.87-1.06 | 0.45 |
| HRP2 | ng/ml | 8415 | 4133-13690.8 |  | 134 | 9470 | 2790-11070 |  | 27 | 1.00 | 1.00-1.00 | 0.92 |
| HIV status | negative |  |  | 84.3 | 127 |  |  | 88.5 | 26 |  |  |  |
|  | positive |  |  | 15.8 |  |  |  | 11.5 |  | 1.43 | 0.39-5.23 | 0.59 |
| **Retinal** | | | | | | | | | | | | |
| Retinal hemorrhages | none |  |  | 27.1 | 133 |  |  | 29.6 | 27 |  |  |  |
|  | 1 to 5 |  |  | 36.8 |  |  |  | 29.6 |  | 1.36 | 0.47-3.97 | 0.57 |
|  | 6 to 20 |  |  | 18.1 |  |  |  | 18.5 |  | 1.07 | 0.31-3.65 | 0.92 |
|  | 21 to 50 |  |  | 6.8 |  |  |  | 7.4 |  | 1.00 | 0.18-5.55 | 1.00 |
|  | >50 |  |  | 11.3 |  |  |  | 14.8 |  | 0.83 | 0.22-3.19 | 0.79 |
| Papilledema | negative |  |  | 72.9 | 133 |  |  | 62.9 | 27 |  |  |  |
|  | positive |  |  | 27.1 |  |  |  | 37.0 |  | 0.63 | 0.26-1.5 | 0.29 |
| Disc hyperemia | negative |  |  | 70 | 130 |  |  | 84.6 | 26 |  |  |  |
|  | positive |  |  | 30 |  |  |  | 15.4 |  | 2.4 | 0.76-7.29 | 0.14 |
| Macular whitening | none |  |  | 9.9 | 132 |  |  | 3.7 | 27 |  |  |  |
|  | <1/3DA |  |  | 30.3 |  |  |  | 44.4 |  | 0.26 | 0.03-2.17 | 0.21 |
|  | 1/3-1DA |  |  | 32.6 |  |  |  | 18.5 |  | 0.66 | 0.07-6.18 | 0.72 |
|  | >1DA |  |  | 27.3 |  |  |  | 33.3 |  | 0.31 | 0.04-2.67 | 0.29 |
| Foveal whitening | none |  |  | 15.9 | 132 |  |  | 15.4 | 26 |  |  |  |
|  | <1/3 fovea |  |  | 38.6 |  |  |  | 46.2 |  | 0.81 | 0.23-2.8 | 0.74 |
|  | 1/3-2/3 fovea |  |  | 16.7 |  |  |  | 19.2 |  | 0.84 | 0.2-3.5 | 0.81 |
|  | >2/3 fovea |  |  | 28.8 |  |  |  | 19.2 |  | 1.45 | 0.35-5.9 | 0.61 |
| Orange vessels, temp periphery | absent |  |  | 62.9 | 108 |  |  | 75 | 20 |  |  |  |
|  | present |  |  | 37.0 |  |  |  | 25 |  | 1.76 | 0.6-5.22 | 0.31 |
| White vessels, temp periphery | absent |  |  | 86.1 | 108 |  |  | 75 | 20 |  |  |  |
|  | present |  |  | 13.9 |  |  |  | 25 |  | 0.48 | 0.15-1.53 | 0.22 |
| White capillaries, temp periphery | absent |  |  | 85.2 | 108 |  |  | 75 | 20 |  |  |  |
|  | present |  |  | 14.8 |  |  |  | 25 |  | 0.52 | 0.17-1.64 | 0.27 |
| Macular capillary non-perfusion | Grade 0 or 1 |  |  | 6.87 | 131 |  |  | 11.54 | 26 |  |  |  |
|  | Grade 2 |  |  | 47.33 |  |  |  | 50.00 |  | 1.59 | 0.38-6.69 | 0.53 |
|  | Grade 3 or 4 |  |  | 45.80 |  |  |  | 38.46 |  | 2.00 | 0.46-8.68 | 0.36 |
| Peripheral CNP | Grade 0 or 1 |  |  | 39.85 | 133 |  |  | 25.93 | 27 |  |  |  |
|  | Grade 2 |  |  | 24.81 |  |  |  | 25.93 |  | 0.62 | 0.2-1.94 | 0.41 |
|  | Grade 3 or 4 |  |  | 35.34 |  |  |  | 48.15 |  | 0.48 | 0.18-1.30 | 0.15 |
| Punctate focal leak | None |  |  | 63.43 | 134 |  |  | 66.67 | 27 |  |  |  |
|  | 1-5 sites |  |  | 28.36 |  |  |  | 33.33 |  | 0.89 | 0.37-2.17 | 0.81 |
|  | >5 sites |  |  | 8.21 |  |  |  | 0.00 |  | - | - | - |
| Large focal leak | None |  |  | 83.58 | 134 |  |  | 77.78 | 27 |  |  |  |
|  | 1 site |  |  | 6.72 |  |  |  | 0.00 |  | - | - | - |
|  | >1 site |  |  | 9.70 |  |  |  | 22.22 |  | 0.41 | 0.14-1.19 | 0.10 |
| Large/small venule leak | None |  |  | 56.39 | 133 |  |  | 46.15 | 26 |  |  |  |
|  | Grade 1 |  |  | 32.33 |  |  |  | 26.92 |  | 0.98 | 0.36-2.68 | 0.97 |
|  | Grade 2 or 3 |  |  | 11.28 |  |  |  | 26.92 |  | 0.34 | 0.12-1.01 | 0.053 |
| Post-capillary venule leak | None or grade 1 |  |  | 75.19 | 133 |  |  | 53.85 | 26 |  |  |  |
|  | Grade 2 |  |  | 17.29 |  |  |  | 26.92 |  | 0.46 | 0.17-1.27 | 0.13 |
|  | Grade 3 or 4 |  |  | 7.52 |  |  |  | 19.23 |  | 0.28 | 0.08-0.94 | **0.04** |
| Disc leak | Absent |  |  | 18.66 | 134 |  |  | 0.00 | 27 |  |  |  |
|  | Present |  |  | 81.34 |  |  |  | 100.00 |  | - | - | - |
| Intravascular filling defect in large arterioles | Absent |  |  | 86.15 | 130 |  |  | 77.27 | 22 |  |  |  |
|  | Present |  |  | 13.85 |  |  |  | 22.73 |  | 0.55 | 0.18-1.67 | 0.29 |
