# Supplementary material for: How Does Blood-Retinal Barrier Breakdown Relate to Death and Disability in Pediatric Cerebral Malaria?
Source: J Infect Dis. 2020 Aug 26;225(6):1070–80. doi: 10.1093/infdis/jiaa541 (PMC8922008; doi:10.1093/infdis/jiaa541)

**Supplementary Table 4. Mediation analysis of causal routes from large focal leak (LFL) to death.** This assumes that LFL is proportionate manifestation of analogous leakage in the brain. One path involves two steps: LFL to severe brain swelling, and severe brain swelling to death; the effect of LFL on death is mediated by severe brain swelling. The other goes directly from LFL to death; LFL (or more precisely, the intracranial analogue represented by LFL) causes death directly. The absence of other connectors between boxes illustrates the assumption that there are no unmeasured exposure-mediator, exposure-outcome, or mediator-outcome confounders. The natural indirect effect describes the effect of the exposure (LFL) on the outcome (death) that operates through the mediator (severe brain swelling). In comparison the natural direct effect represents whatever effect would remain after disabling the path between the exposure and the mediator.

|  | Estimate | Standard error | P | 95% CI | n |
| --- | --- | --- | --- | --- | --- |
| Controlled direct effect | 1.60 | 0.65 | 0.47 | 0.45 to 5.66 | 133 |
| Natural direct effect | 1.60 | 0.65 | 0.47 | 0.45 to 5.66 | 133 |
| Natural indirect effect | 2.04 | 0.30 | **0.02** | 1.14 to 3.66 | 133 |
| Marginal total effect | 3.26 | 0.67 | 0.08 | 0.87 to 12.22 | 133 |


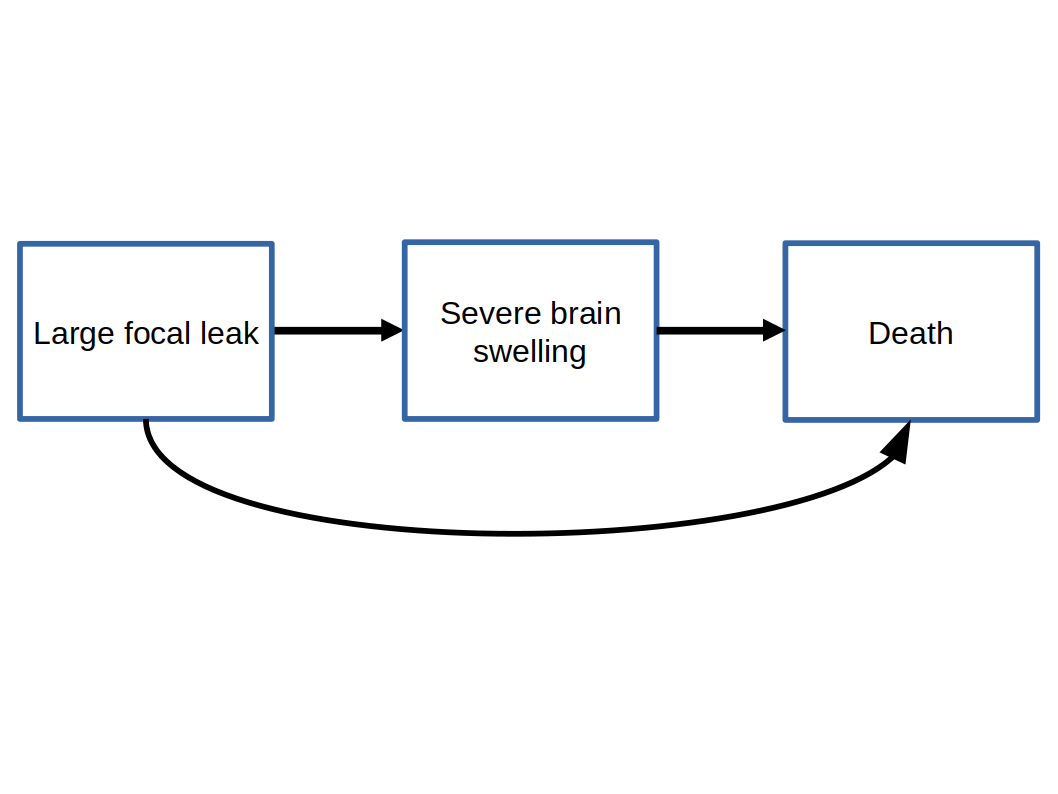

Supplement: jiaa541_suppl_Supplementary_Table_4 [file jiaa541_suppl_supplementary_table_4.docx]
