# Supplementary material for: How Does Blood-Retinal Barrier Breakdown Relate to Death and Disability in Pediatric Cerebral Malaria?
Source: J Infect Dis. 2020 Aug 26;225(6):1070–80. doi: 10.1093/infdis/jiaa541 (PMC8922008; doi:10.1093/infdis/jiaa541)
Supplement: jiaa541_suppl_Supplementary_Figure_Legends [file jiaa541_suppl_supplementary_figure_legends.docx]

**Supplementary Figure 1. Derivation of cohorts.**

A) Entire fluorescein angiogram cohort. B) Fluorescein angiogram and MRI brain subset cohort. Ret- malarial retinopathy absent; Ret+ malarial retinopathy present

**Supplementary Figure 2. Capillary non-perfusion and leakage**

A) Admission color and fluorescein angiogram (FA) images with retinal whitening and peripheral capillary non-perfusion (CNP) and no leakage. Note the attenuated venule in center is orange, with intravascular filling defects on FA. B) Day 1 showing development of larger zones of CNP and fluorescein leakage from vessels crossing or adjacent to non-perfused zones. C) Day 2 showing improvement of CNP (re-perfusion) and leakage from re-perfusing vessels.

**Supplementary Figure 3. Histopathology of monocytes and hemozoin.**

A) Hemozoin-laden monocytes identified in the core of white-centered hemorrhages by the presence of dark brown malaria pigment and typical kidney-shaped nuclei. Cells are marked by arrows. Anti-CD45 immunohistochemistry (red) and hematoxylin (blue) counterstaining are shown. Scale bar = 20 µm. B-D) Characterization of monocytes and hemozoin by hematoxylin and eosin staining. B) Monocytes with phagocytosed hemozoin in capillaries. Scale bar = 10 µm. C) Monocytes in venules. Scale bar = 20 µm. D) Extra-erythrocytic hemozoin in retinal capillaries. Scale bar = 10 µm.
